# Rational design of non-resistant targeted cancer therapies

**DOI:** 10.1038/srep46632

**Published:** 2017-04-24

**Authors:** Francisco Martínez-Jiménez, John P. Overington, Bissan Al-Lazikani, Marc A. Marti-Renom

**Affiliations:** 1 CNAG-CRG, Centre for Genomic Regulation (CRG), Barcelona Institute of Science and Technology (BIST), Baldiri i Reixac 4, 08028 Barcelona, Spain; 2 Gene Regulation, Stem Cells and Cancer Program, Centre for Genomic Regulation (CRG), Dr. Aiguader 88, 08003 Barcelona, Spain; 3 Universitat Pompeu Fabra (UPF), Barcelona, Spain; 4 Medicines Discovery Catapult Block 35, Mereside, Alderley Park, Alderley Edge, Cheshire, SK10 4TG, UK; 5 The Institute of Cancer Research, London, UK; 6 ICREA, Pg. Lluís Companys 23, 08010 Barcelona, Spain

## Abstract

Drug resistance is one of the major problems in targeted cancer therapy. A major cause of resistance is changes in the amino acids that form the drug-target binding site. Despite of the numerous efforts made to individually understand and overcome these mutations, there is a lack of comprehensive analysis of the mutational landscape that can prospectively estimate drug-resistance mutations. Here we describe and computationally validate a framework that combines the cancer-specific likelihood with the resistance impact to enable the detection of single point mutations with the highest chance to be responsible of resistance to a particular targeted cancer therapy. Moreover, for these treatment-threatening mutations, the model proposes alternative therapies overcoming the resistance. We exemplified the applicability of the model using EGFR-gefitinib treatment for Lung Adenocarcinoma (LUAD) and Lung Squamous Cell Cancer (LSCC) and the ERK2-VTX11e treatment for melanoma and colorectal cancer. Our model correctly identified the phenotype known resistance mutations, including the classic EGFR-T790M and the ERK2-P58L/S/T mutations. Moreover, the model predicted new previously undescribed mutations as potentially responsible of drug resistance. Finally, we provided a map of the predicted sensitivity of alternative ERK2 and EGFR inhibitors, with a particular highlight of two molecules with a low predicted resistance impact.

Non-selective cytotoxic agents have traditionally dominated cancer treatment. However, the strong side effects and the limited effectiveness associated with drug resistance have led to the search of alternative treatments^1^. In the last decade, rationally designed ‘targeted’ therapies have been developed as less damaging and more accurate alternative to treat cancer^2^. In fact, targeted therapies have produced substantial clinical responses in the treatment of chronic myeloid leukemia (CML)^3^, non-small cell lung cancer (NSCLC)^4^ and melanoma^5^. Unfortunately, after initial good response to targeted therapies, tumors develop resistance to these treatments causing disease relapse^6^,^7^. Many of these targeted therapies interfere with cell-signalling pathways, and in particular target members of the protein kinase gene family^8^.

There are several mechanisms conferring drug resistance to targeted therapies^9^. Mechanisms such as activation of survival signaling pathways, or the inactivation of downstream death-signaling pathways^10^,^11^, increasing drug efflux or alterations in drug metabolism^12^,^13^. Epigenetic changes and their influence of in the tumor microenvironment have also been proposed to play a role in chemoresistance^13^,^14^. Moreover, secondary mutations of drug targets are frequently reported as a mechanism of drug resistance. In NSCLCs, patients initially responding to first generation EGFR inhibitors such as gefitinib and erlotinib, typically acquire resistance within 1 year. In 50% of such cases, a secondary T790M gatekeeper mutation has been identified^15^,^16^. Recently, a third generation EGFR inhibitors that specifically bind to T790M-EGFR, such as rociletinib^17^ or osimertinib^18^ have been designed to overcome resistance in EGFR-T790M positive patients^19^.

Unfortunately, EGFR-T790M is a single example, we still are far from completely overcoming the clinical challenge of resistance due to mutations in oncogenic kinases. Many studies have been carried out to both systematically analyze resistance to kinase inhibitors^20^ and to propose alternatives to standard kinase inhibitor treatments^21^. Nevertheless, these studies do not cover the whole spectrum of possible mutations of the target, being usually limited to a small, and clinically reported, number of kinase mutations. Moreover, the nature and *in situ* evolution of tumors is complex and heterogeneous^22^. Estimates of the number of coding mutations in the entire cell population of a tumor are of the order of thousands or even millions of mutations depending of the tumor type and size^23^. Standard NGS sequencing of solid biopsies only enables the detection of mutations present in >5% of tumor cells^24^. The low sensitivity of standard NGS technologies alongside the heterogeneous nature of solid tumors, may lead to a significant loss of low-frequency mutations present in small cell number populations. Remarkably, low-frequency mutations can confer resistance to targeted therapies and therefore, become clonal drivers once the cancer treatment begins^7^,^25^,^26^. There is a clear need for a method that can prospectively predict the likelihood of specific drug-resistance mutants arising to enable the pre-emptive screening for these mutants in patients and the design of drugs that can overcome them.

The invasive nature and the technical limitations associated with sequencing methods of solid biopsies highlight the importance of computational models in cancer evolution and drug resistance. The advent of the massive cancer genomic data has prompted the development of several mathematical and computational models^27^. Some of these models focus on characterizing tumor evolutionary processes^28^,^29^,^30^ while others, study tumor response to single targeted treatment^31^,^32^,^33^,^34^ or combinational therapy^35^. However, none of these models, which are usually applied to known drug-resistant mutations, specifically predict which are the causative mutations leading to drug resistance.

Here we present a general computational framework for the *de-novo* prediction of coding mutations with the potential to confer specific resistance to small molecule targeted therapies. Additionally, the model provides a list of alternative compounds/drugs ranked by their predicted sensitivity to these *resistance-like* mutations. The framework connects the tumor type-specific mutational landscape of tumors with the drug-resistance phenotype generated by spontaneous mutations in drug targets. We exemplified the applicability of the framework in two protein kinases, EGFR and ERK2 (also known as MAPK1). EGFR is well-studied model in resistance to targeted cancer therapy, and consequently, is a good system to validate the full scope of the framework. We computationally predict the likelihood and the resistance impact of specific EGFR residues involved in the binding of gefitinib in LUAD and LSCC. Additionally, using the mutational signatures previously defined^36^, we also analyzed the possible aetiology (or aetiologies) associated to each of the most critical and possible to occur EGFR mutations. Our model correctly predicts the phenotype of the EGFR-T790M mutation, with the added value of the identification of new previously undescribed mutations that may confer resistance to gefitinib treatment. ERK2, on the other hand, is a promising target in the treatment of melanoma^37^,^38^ and colorectal cancer^39^. We predict the VTX11e-resistance potential of 424 potential ERK2 mutations. These predictions include the correct identification of eight mutations alongside new unseen ERK2 mutations predicted to confer resistance to VTX11 treatment in melanoma and colorectal cancer. Moreover, the structural nature of the predictions helped to elucidate the specific mechanism of resistance of each mutation. Finally, for both EGFR and ERK2 treatment-threatening mutations, the model proposed alternative inhibitors that might overcome resistance.

## Methods

### The likelihood model

**Figure 1:**
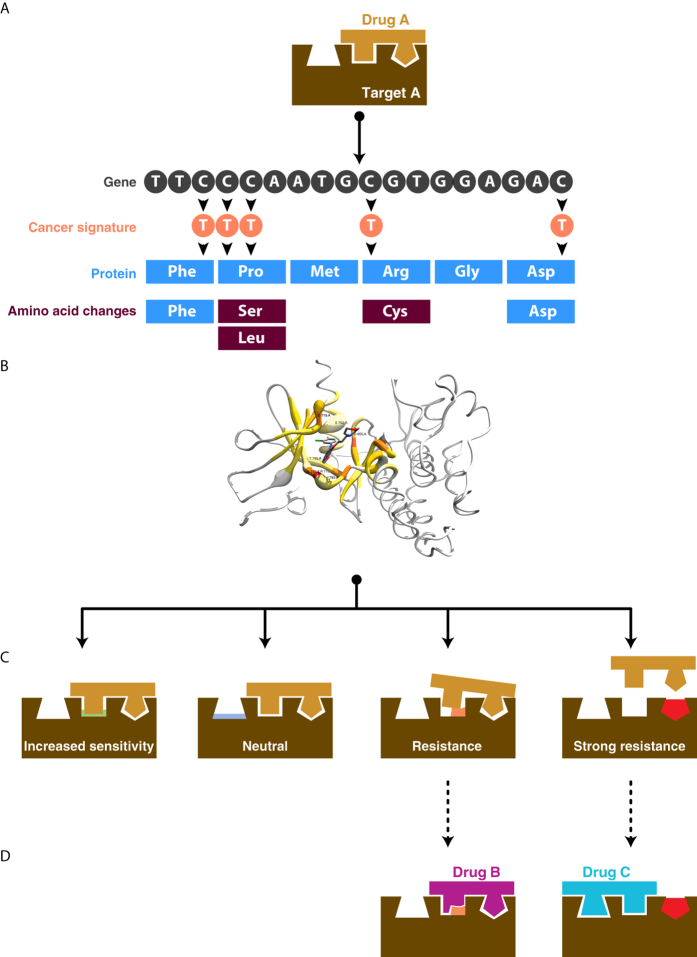
Schematic representation of the developed framework. (**A**) For a particular targeted cancer therapy, the most likely mutations of the protein target are defined using the mutational signatures associated with that cancer class^36^. (**B**) 3D models of the mutations in the target structure are generated using the MODELLER package. (**C**) Structural and sequential information of the 3D-mutant models is used by a Random Forest Classifier (RFC) to predict the resistance potential of these mutations. (**D**) For the mutations classified as resistance-like, the model proposes alternative non-resistant compounds/drugs that may skip resistance.

We developed a model to estimate cancer-associated likelihoods of spontaneous mutation in arbitrary drug targets (Fig. 1). First, using published mutational signatures^36^,^40^, we annotated the contribution of each of the 30 signatures to the 36 different classes of cancer present in the study. Second, for each signature, we extracted the probabilities of the 96 possible pyrimidine-based mutations (C > A, C > T, C > G, T > A, T > C, T > G) in their 5′ and 3′ contiguous bases context from the COSMIC database (from http://cancer.sanger.ac.uk/cosmic/signatures). Next, for each signature without described strand-bias we extended the probabilities to the purine-based mutations (G > A, G > C, G > T, A > C, A > T, A > G). Signatures with strong mutational strand-bias towards a specific type of base pair were manually updated depending of their specific type of bias. For instance, signature 7 has a strong transcriptional strand-bias indicating that mutations occurs a pyrimidines base pairs, therefore the mutational probabilities of purines in signature 7 are set to 0. Signatures with strong mutational strand-bias are signatures 4, 7, 11, 22, 24 and 29. This approach resulted in a total of 192 mutational probabilities for each signature.

We compute the likelihood (*L*_*m,c*_) of a specific mutation (*m*) in a particular type of cancer (*c*) as the sum the probabilities of that mutation in all the signatures involved in that cancer type, weighted by the specific contribution of that signature to the cancer class. Since, several nucleotide mutations can lead to the same amino acid change (i.e. are synonymous), all these probabilities are eventually added to measure the amino acid mutation likelihood using the Eq. 1:


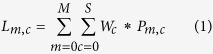


where *M* is all possible nucleotide changes associated to an amino acid mutation *m, S* are the signatures associated to the studied cancer class *c, W*_*c*_ is the contribution of signature *c* to the studied cancer and *P*_*m,c*_ is the probability of a given mutation *m* in the signature *c*.

### EGFR and ERK2 mutants and structural model generation

We applied the likelihood model to predict the probability of mutation of all the amino acids involved in the EGFR binding site to gefitinib (PDB code: 4WKQ), and VTX11e binding to ERK2 (PDB code: 4QTE). We defined a drug binding-site in a protein structure as all the amino acids with at least one atom within 9.5 Å to the co-crystalized ligand.

Next, models of all the possible mutations of the drugs binding-sites were generated using the *mutate_model* function of the MODELLER software with default parameters^41^,^42^. Due to the fact that the produced 3D model is generated for single amino acid mutation, it is highly likely to be accurate^43^. Models for truncating mutations (*i.e.,* introducing a stop codon) were not generated. The final number of three-dimensional (3D) models was 367 and 424 for EGFR and ERK2, respectively.

### Enrichment analysis of the predicted nucleotide mutations likelihood

To measure whether a nucleotide mutation A > B is enriched among the most likely target mutations in a particular cancer class, we calculated the odds ratio of the specific nucleotide mutation A > B for the top 50 likely mutants. More specifically, the odds ratio of a particular nucleotide mutation *A* > *B* at the *i*^*th*^ position in the distribution is given by Eq. 2:


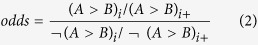


where (*A* > *B*)_*i*_ denotes the number of *A* > *B* mutations between the *0* and *i*^*th*^ position. (*A* > *B*)_i+_ represents the number of A > *B* mutations between *i* + *1* and the *N*^*th*^ position, being N the total number of amino acid mutations.

### Drug-response predictor

We developed two Random Forest Classifiers (RFC). The first classifier, called aa-RFC (amino acid based RFC) predicts the phenotypic effect of an amino acid mutation to the binding affinity between a drug and the target protein. The second classifier, called lig-RFC (ligand based RFC), aims to predict the sensitivity of a group of compounds to a particular mutation in their protein target. Both classifiers use structural and sequence information of the drug-protein interaction to perform the predictions (see below for detailed information about the specific features used for each classifier). The lig-RFC emphasizes in the ligand-target interaction while omitting some information relative to the amino acid characteristics, which makes it computationally faster to build. Both classifiers were built using the WEKA package^44^ with the following parameters: *numTrees* = 1,000; *numFeatures* = 20; *maxDepth* = FALSE. Evaluation of the classifiers performance was done by 10-fold cross validation (CV). Additionally, the relative importance of each variable in the classifiers was calculated by the *randomForest* package of R^45^. Next, we describe all necessary steps to generate and test the classifiers.

### Dataset generation

The aa-RFC and lig-RFC models were trained using the Platinum database^46^. Briefly, this database contains information about experimentally measured changes in drug binding affinity upon mutations. Moreover, most the entries in the database contain crystal structures of the drug-protein complexes. When no crystal structure was available for either the wild-type or the mutated structure, a 3D model was generated using MODELLER with default parameters. The database originally included 1,008 instances. Since the aa-RFC classifier has been developed to individually assess the resistance potential of a single mutation, we removed 208 instances containing double (155), triple (30) or more mutants. The final dataset contained 770 instances, including 377 PDB entries and 584 3D models. Next, the database was split into four different classes corresponding to four different phenotypes: (i) “strong resistance” (SRES, 293 instances) with a 5-fold or greater drop in binding affinity, which disrupt the binding of the compound with the target protein; (ii) “resistance” (RES, 227 instances) with between a 5- and 1.2-fold drop in affinity; (iii) “neutral” (NEU, 70 instances) with between a 1.2-fold drop and 1.2-fold increase in affinity, which indicates not significant alteration of the binding affinity of the compound; and (iv) “increased sensitivity” (ISEN, 180 instances) with a 1.2-fold or greater increase in the affinity of the compound. Finally, the unbalanced nature of the dataset could have introduced bias in the classifier predictions towards SRES and RES classes because of the higher number of instances. Therefore, we randomly removed instances of the SRES and RES classes to reduce the number of data points used in model training to 180. The final dataset was therefore composed by 180 instances of the SRES, RES and ISEN classes and 70 of the NEU class.

### Sequence and structure features calculated from the 3D models/structures

For each instance in the dataset we calculated a set of features to describe the structural and sequential changes introduced by the mutation. The complete list of features alongside their description and information about their inclusion in the two classifiers are next detailed:

1. **Molecular surface area of the drug binding-site (aa-RFC, lig-RFC).** Total molecular surface area of wild type (WT) and mutated (MT) drug binding-site. Additionally, the absolute numerical difference between the two values was included. The *get*_*area* function of PyMol 1.8 Version^47^ was used for their calculation.
2. **Solvent accessibility of the WT and MT amino acid (aa-RFC, lig-RFC).** Additionally, the absolute numerical difference between the two values was included. The *get*_*area* function of PyMol 1.8 Version^47^ was used for their calculation.
3. **Relative solvent accessibility (RSA) of the WT/MT residue (aa-RFC, lig-RFC).** Ratio between the solvent accessibility area and the general residue surface area calculated using DSSP with default parameters^48^. Additionally, the absolute numerical difference between the two values was included
4. **Half sphere exposure of the WT/MT amino acid (aa-RFC)**^49^. The *HSExposure* class from the *Biopython* library^50^ was used for its calculation. Additionally, the absolute numerical difference between the two values was included.
5. **Type of amino-acid change (aa-RFC, lig-RFC)**. A vector of 20 positions representing the 20 amino acids. In the vector, a −1 represents the wild type amino acid, a 1 represents the new residue introduced by the mutation, and 0 represents no change.
6. **Hydrogen bonding (aa-RFC, lig-RFC).** We calculated whether there is a hydrogen bond between the WT/MT residue and the drug bound molecule. Information about the hydrogen bond type and distance were also included. The upper bound to assess the presence of a hydrogen bond was 3.2 Å.
7. **Structural environment of the amino acid (aa-RFC, lig-RFC).** We represented the structural environment with concentric spheres surrounding the mutated amino acid. Each of the spheres has different radius ranging from 0 Å to 6 Å in steps of 1 Å. The spheres were represented using 6 vectors of 20 positions indicating the presence or absence of an amino acid. A number one in a vector implied that the amino acid representing that position was within that radius.
8. **Sequence environment (aa-RFC, lig-RFC).** We defined the amino acid sequence environment as the composition of all 10 contiguous amino acids in sequence (5 amino acids preceding and 5 amino acids following the mutated amino acid). Each position was represented by a vector of 20 amino acids where 1 indicated presence and 0 absence of the given amino acid in the sequence environment.
9. **Secondary structure of the amino acid (aa-RFC, lig-RFC)**. We calculated the secondary structure of the WT/MT amino acid using DSSP with default parameters^48^.
10. **Protein stability change (aa-RFC, lig-RFC)**. We calculated the change in the stability of the protein caused by the mutation using I-Mutant 2.0^51^. We included two variables, the first one describes the numerical change in stability measured in kcal/mol and the second was a categorical variable representing the sign of stability change: UNSTABLE for negative values, STABLE for positive values and UNKNWON for mutations where I-Mutant 2.0 could not compute a score (that is, in 19% of cases).
11. **Residue conservation (aa-RFC, lig-RFC).** To calculate the conservation score we first performed a BLAST search^52^ using as query the target sequence. The resulting multiple alignment was used as input to the *SubsMat* function from Biopython library^50^ to obtain a residue conservation score based on the BLOSUM62 matrix^53^.
12. **Structural alignment of the MT model to the WT structure (aa-RFC, lig-RFC).** Root Mean Squared Deviation (RMSD) of the structural alignment between the wild-type and the mutated protein structures. Two different RMSD were calculated, the first resulted from the original structural alignment and the second from the refined one. The *Super* function from PyMol 1.8 Version^47^ was used to perform both structural alignments.
13. **Distance to the ligand (aa-RFC, lig-RFC).** We measured the distances between the alpha carbon of the WT/MT amino acid to all the atoms of the ligand. Next, we calculate the minimum, maximum and average distances to the ligand. For all of these distances the absolute numerical difference between the WT and MT value was included. PyMol 1.8 Version^47^ was used for their calculation.
14. **Charge of the WT and MT amino acids (aa-RFC, lig-RFC).** A vector of 20 positions was generated with −1 for negatively charged amino acids (ASP, GLU), a + 1 for positively charged amino acids (LYS, ARG) and 0 for the remainders.
15. **Change in the hydrophobicity (aa-RFC, lig-RFC).** We calculate the difference between WT and the MT amino acids using a pre-calculated hydrophobicity scale^54^.
16. **Drug affinity of the ligand with the WT protein (aa-RFC, lig-RFC)**. We retrieved the binding affinity using BindingDB^55^. Depending of the availability on the BindingDB record, the binding affinity was measured by the inhibitory constant (Ki), the dissociation constant (Kd) or the half maximal inhibitory concentration (IC_50_) measures.
17. **Salt bridge between WT/MT amino acid with other residues (aa-RFC)**. Number of salt bridges between the GLU and ASP amino acids of the WT/MT protein surface were calculated. Additionally, the absolute numerical difference between the two values was included. An upper bound cu-off of 4.0 Å distance between the anionic group of GLU/ASP and the cationic group of LYS/ARG was used.
18. **Salt bridge between WT/MT amino acid with the ligand (aa-RFC, lig-RFC)**. We used the PLIP^56^ software with default parameters (v1.2.0) to calculate salt bridges between ASP or GLU residues of the protein and the query drug. Information about the distance measured in Å, type of acceptor and donor groups (Phosphate, Carboxylate, Guanidine, Tertamine or Quartamine) was also included in the lig-RFC.
19. **Disulphide bonds (aa-RFC).** If the mutated residue is a cysteine, we identified putative intra-cysteine disulphide bonds. The expected SG–SG distance for disulfide bond is around 2 Å but more generous definition accounts for inaccuracies in experimental data. Therefore we used disulphide bond distances between 1.8 Å and 2.2 Å.
20. **Halogen bonds (lig-RFC).** The presence of halogen bonds between the WT/MT amino acid and the ligand. It also included information about the type of donor and acceptor atoms. This feature was calculated using PLIP^56^ with default parameters (v1.2.0). Features 21 to 24 were also obtained using PILP.
21. **π-stacking interactions (lig-RFC).** The presence of π-stacking interactions between the ligand and the WT/MT residue including information about the distance and group of interactions.
22. **π**-**cation interactions (lig-RFC).** The presence of π-cation interactions between the ligand and the WT/MT residue including information about the distance and atoms group involved in the interactions.
23. **Water bridges (lig-RFC).** The presence of water bridges between the WT/MT amino acid and crystallized waters molecules including the type of donor and acceptor atoms.
24. **Hydrophobic interactions (lig-RFC).** The presence of hydrophobic interactions between the ligand and the WT/MT amino acid including information about the distance of the interaction.

In summary, a total of 58 features were used for the aa-RFC and a total of 89 were used for the lig-RFC. The complete list of features and values for the training set is available as supplementary file.

### Predictions and resistance score

We applied the aa-RFC to individually predict the phenotype of each of the EGFR and ERK2 mutations defined by the likelihood model. For each compound-protein-mutant, the aa-RFC assigns a confidence score for each the four possible phenotypes (SRES, RES, NEU and ISEN classes). The class-confidence scores addition is equal to 1. The highest class-confidence score corresponds to the predicted class. Next, we defined a global Resistance Score (RS) as the sum of the SRES and RES scores minus the ISEN and NEU weighted by the precision of each class in the aa-RFC training. The normalized RS measure aims at assessing the resistance impact of a mutation in a target for the studied drug. The RS score is defined as (Eq. 3):


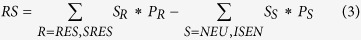


where *R* are the two classes of resistance (*i.e.,* SRES and RES) and *S* are the two classes of non-resistance (*i.e.,* NEU and ISEN), *S*_*x*_ is the aa-RFC confidence score for the class *x* and *P*_*x*_ is the global aa-RFC accuracy for the class *x* after a 10-fold cross validation. Finally, a normalized RS (NRS) was calculated by scaling all RS values within an experiment between 1.0 (that is, the highest RS) and 0.0 (that is, the lowest RS).

### Creating a dataset of insensitive molecules

To identify compounds that may result insensitive to a particular mutation, and thus be alternative to a given treatment, we first manually extracted all compounds reported the Food and Drug Administration (FDA, http://www.fda.gov) and the National Cancer Institute (NCI; http://www.cancer.gov) web sites that modulate the studied protein target. Second, we collected all co-crystallized molecules with the protein target. Next, molecules with no experimentally measured binding affinity in BindingDB^55^ were discarded. Due to the limited number of small molecules co-crystallized with ERK2, we extended the search to small-molecule ERK2 inhibitors with IC_50_ better than (or equal to) 100 nM from the ChEMBL database^57^. Finally, we manually included other compounds of interest into the final dataset, which resulted in a total of 19 and 75 possible non-resistant molecules to EGFR and ERK2 respectively.

### Predicting molecules likely to be insensitive to a binding site mutation in the protein target

Once the dataset was built, we used it to identify molecules whose affinity may not decrease by a mutation in the protein target. Depending of the source of the molecule, the methodology to assess the sensitivity was different: (i) for the two first subsets (*i.e.,* those co-crystallized with the target) we defined the potential of the mutation to confer resistance using the crystal structure of the drug bound to the target; (ii) for those molecules extracted from ChEMBL and those manually included, we selected the top-ranked pose by Autodock Vina^58^ by performing docking between the compounds and the target binding pocket. In both cases, each compound-target-mutation prediction was further scored by the normalized RS.

### Predicting changes in affinity using AutoDock Vina

Finally, to assess the base-line accuracy when no additional information is given, a new classifier was trained using only the calculated binding affinity change by AutoDock Vina. For each wild-type and mutated complex in the aa-RFC training set, we first calculated the predicted affinity of the top ranked pose by AutoDock Vina. Next, the two affinities were passed to a RFC classifier that predicted the phenotypic class of the instance. The classifier parameters and the subsequent validation were performed using the same parameters than in the aa-RFC training. For each instance in the training set the fold change in the predicted affinity was calculated as the ratio between the wild type and the mutated predicted affinities.

## Results

### Prediction of the drug binding affinity change upon single mutation

**Figure 2:**
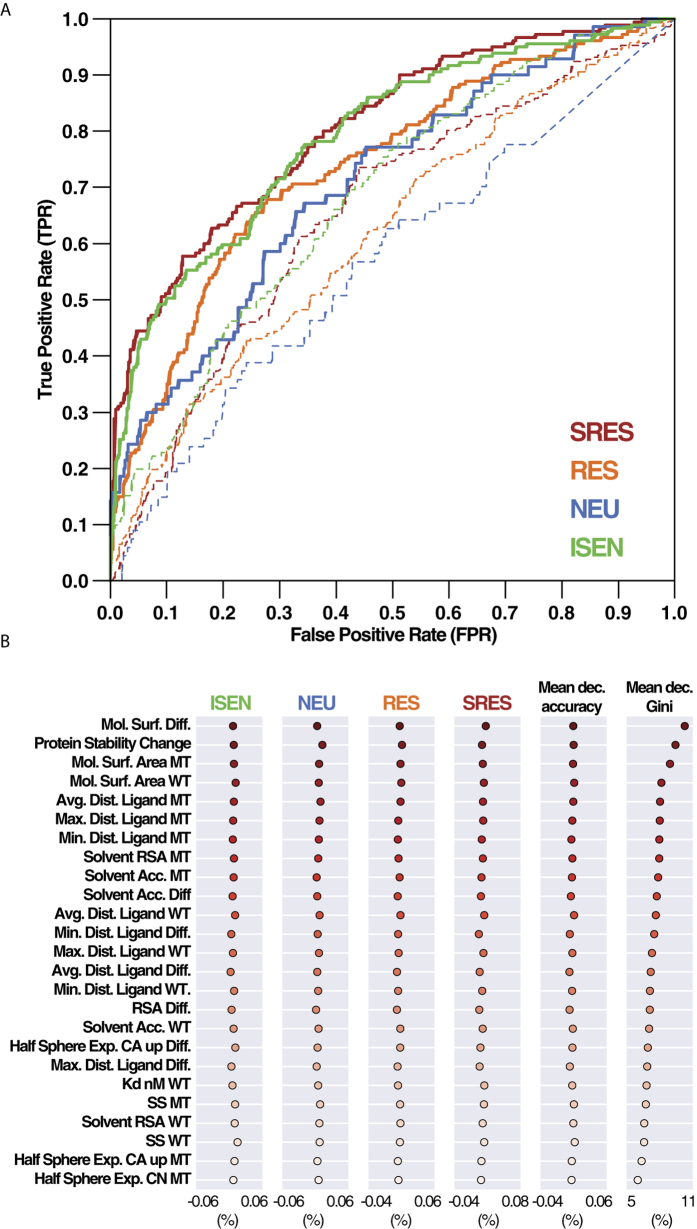
RFC accuracy. (**A**) Receiver operating characteristic (ROC) curves of the four phenotypic classes (that is, strong resistance “SRES”, resistance “RES”, neutral “NEU” and increased sensitivity “ISEN”) after 10-fold cross validation. Solid lines correspond to the results of our RFC classifier; dashed lines correspond to the results of a non-trained approach based on the AutoDock Vina results. (**B**) Relative importance of the top 25 most informative variables used by the aa-RFC. Features are ranked by the mean decreased Gini score based on the Gini impurity index^60^. The rest of aa-RFC features are not shown for clarity.

We tested the performance of the aa-RFC classifier using the Platinum database^46^. The average AUC of the classifier (0.77) together with a Kappa statistic of 0.40 59 indicated an overall high accuracy of the classifier, especially considering that this is a four-class classifier (Fig. 2A). The SRES class was the best predicted with a 0.81 AUC (0.63 precision and 0.62 recall). The second best predicted class was the ISEN class with a 0.79 AUC (0.59 precision and 0.55 recall). Despite the fact that these two classes performed similarly, the lower recall of the IS class indicated that this class had a higher number of false negatives (FN; *i.e.,* instances of the ISEN class misassigned to another class). This suggested that the classifier might have some difficulties in correctly finding the ISEN true positives (TP; *i.e.,* instances of the ISEN class correctly predicted). More specifically, of the total 180 ISEN instances, 43 were miss-classified as RES, 28 as SRES and 8 as NEU. Overall, the aa-RFC classified tended to over-assign instances to the RES class, which reflected to its performance metrics (0.74 AUC, 0.50 precision and 0.62 recall). Despite of this, it is notable that the aa-RFC resulted in a 0.50 precision for the RES class, which is twice the random value in a four-class classifier. Finally, the NEU class was the worst performing class (0.70 AUC, 0.48 precision, and 0.24 of recall). The low recall value (only one out of four NEU instances were assigned to the class) could be explained by the under-representation of the NEU instances in the training set (only 80 available instances, versus 180 instances of the other classes).

To our knowledge this is the first classifier that predicts the resistance-associated phenotype of a mutation for a drug or drug-like compound binding to a protein. However, there are multiple methods that predict the binding affinity of a drug-protein complex. These methods can be also applied to predict how a mutation can change the binding affinity of a particular binding compound. One of the most extensively used virtual screening methods is AutoDock Vina (ADV)^58^. Overall, the performance of the ADT classifier was worse, with an average AUC of 0.64 (0.77 of the A-RFC) and a Kappa statistic of 0.19 (Fig. 2A). More specifically, the four phenotypic classes had considerably lower AUC values for the ADV predictions. The SRES class resulted in the greatest AUC drop compared to aa-RFC (0.81 to 0.65), followed by the NEU class (0.69 to 0.57), the ISEN class (0.79 to 0.68) and by the RES class (0.71 to 0.63). The individual values in change of affinity for each of the training cases showed that only 13 (1.7%) instances had fold changes greater than 1.2, which suggests that virtual docking methods may have difficulties detecting large changes in affinity upon single mutation.

To assess the contribution of each of the 58 input variables to the aa-RFC classified, we sorted them by their mean decrease Gini^60^, which describes how much each variable contributes to the homogeneity of the nodes and leaves in the resulting random forest. The most informative features were those associated with the change in the molecular surface area and solvent accessibility of the mutated amino acid (ranking positions 1^st^, 3^rd^, 4^th^, 8^th^–10^th^, Fig. 2B). Change in the protein stability measured by I-Mutant 2.0^51^ was ranked in the second position. Multiple measures of the distance from the amino acid to the ligand were ranked from the 5^th^ to the 7^th,^ positions, while other features such as the affinity of the wild type complex (20^th^) or the type of secondary structure of the amino acid (21^st^ and 22^nd^) occupied the following positions. Features based in biochemical properties of the mutated amino acid were clearly overrepresented within the top 25 set (18 out of 25). Only the distance to the ligand and the wild type experimentally measured affinity were included within the top 25 features. Overall, these results showed that the classifier weighted more features based on biochemical properties of the amino acid while gave less relevance to those extracted from specific interaction with the ligand.

### EGFR predicted mutational landscape in LUAD and LSCC cancer types

**Figure 3:**
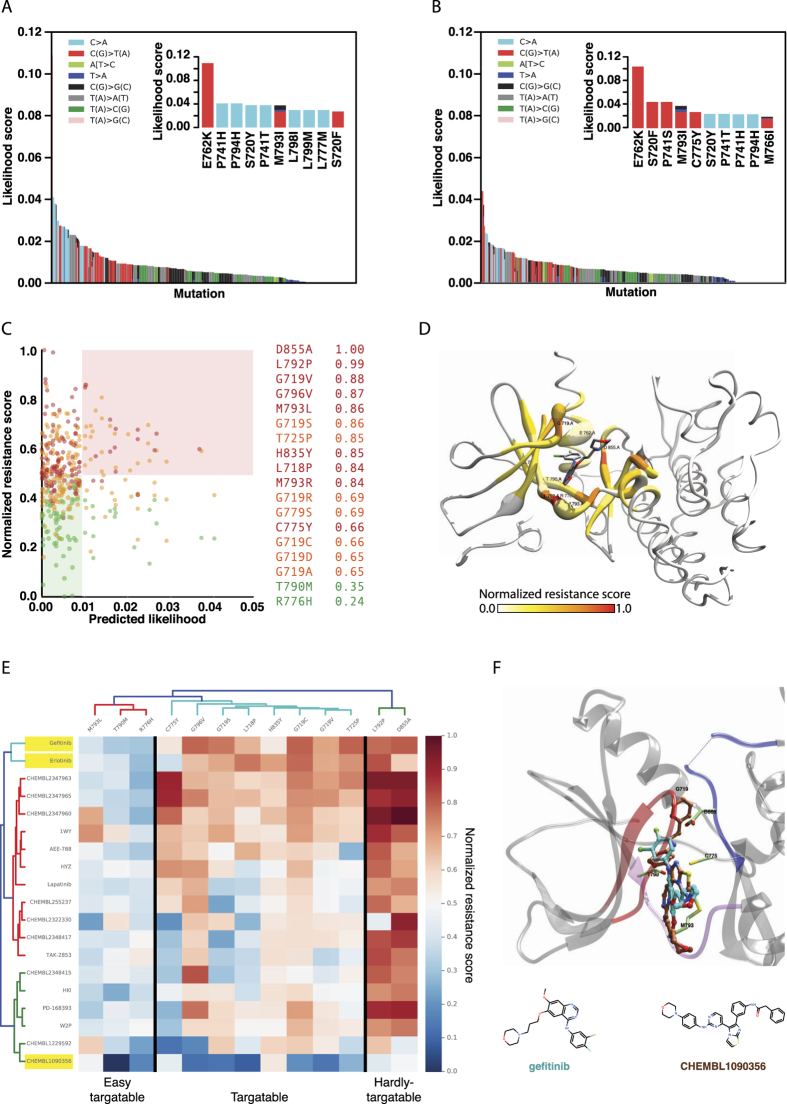
(**A**) Predicted cancer associated-likelihood of mutations in the EGFR binding site for gefitinib for LUAD. Bar high indicates the likelihood of an amino acid mutation and its color the type of nucleotide change that leads to the amino acid mutation. When several nucleotide mutations lead to the same amino acid change, the probabilities were stacked. Inner sets show the top 10 likely mutations. (**B**) Predicted cancer associated-likelihood of mutations in the EGFR binding site for gefitinib for LSCC. Representation as in panel A. (**C**) Predicted likelihood and normalized resistance score (NRS) for EGFR mutations in the binding site of gefitinib for the LUAD cancer type. Each mutation is represented by a dot, which color indicates the predicted class by the aa-RFC (SRES in dark red, RES in orange, NEU in blue and ISEN in green). The red area encompasses mutations with predicted likelihood higher than the median value of all mutations and NRS higher than 0.5. The green area encompasses mutations predicted likelihood lower than the median value of all mutations and NRS lower than 0.5. The top ten resistance mutations, along with other mutations mentioned in the text are listed ordered by their NRS. (**D**) LUAD mutation likelihood and normalized resistance score (NRS) in the 3D structure of EGFR-gefitinib complex (PDB: 4WKQ). The thickness of the ribbons indicates the accumulated mutational likelihood for that particular amino acid. The color represents the accumulated NRS score. Ligands are displayed as sticks. Mutations of amino acids beyond the binding site of the compounds were not considered. (**E**) Predicted sensitivity map for EGFR mutations in the binding site of gefitinib. Columns represents mutations, rows represent the screened compounds. The colour of the cells represents the predicted NRS by the lig-RFC. Name of the compounds are either the generic names for FDA approved drugs or drugs in clinical trials, the ChEMBL accession codes or the PDB accession code for those compounds lacking of an entry in ChEMBL. Compounds mentioned in the text are highlighted with a yellow background. (**F**) Structural mapping of the predicted resistance mutations in the wild type EGFR interaction with gefitinib (cyan) and CHEMBL1090356 (brown). PDB entries: 4WKQ and 3LZB for gefitinib and CHEMBL1090356 respectively. Side chains of the most important contributors to the binding are shown as sticks. The P-Loop is coloured in red, the hinge region in purple and A-Loop in blue.

We studied the mutational probability landscape of EGFR in two different non- NSCLC cancer types: LUAD and LSCC (Fig. 3A and B). The analysis of the mutational landscape indicated that each cancer type had their own underlying molecular mechanisms generating nucleotide changes. Only 20 mutations (that is, ~5% of all binding-site mutations) were ranked in the same position in both cancer types and none of them had the same predicted likelihood. The main discrepancy may be associated to the contribution of signatures 4 and 5 (Supplementary Material). On the one hand, signature 4 is mainly characterized by C > A transversions caused by tobacco smoking^61^. LUAD has a slightly higher contribution from signature 4, resulting in 1.6 times greater average likelihood of C > A mutations in LUAD (0.0226 ± 0.0091 average estimated probability of mutation) than LSCC (0.0143 ± 0.0053). On the other hand, signature 5 has an unknown aetiology and it is associated with T > C substitutions at ApTpN context. Since the signature 5 contribution to LSCC is higher than to LUAD, it resulted in a 2.7 higher average likelihood of the ApTpN mutations in LSCC (0.0057 ± 0.0025) compared to LUAD (0.0037 ± 0.0021).

Analysis of the type of nucleotide change of the top likely mutations revealed an enrichment of C > A mutations in both cancer classes. The highest odds ratio of C > A mutations corresponded to position 21, with an odds ratio value of 16.2 and position 29^th^ with an odds ratio value of 10.0 in LUAD and LSCC, respectively. Additionally, seven (P741H, P794H, S720Y, P741T, L798I, L799M and L777M) and four (S720Y, P741T, P741H and P794H) mutations within the top 10 were C > A mutations in LUAD and LSCC, respectively (inner sets in Fig. 3A and B). An exception to this trend was the top likely mutation, that is E762K, caused by T[G > A]A (T[C > T]A in pyrimidine base pair) mutation. This mutation was associated to signature 2, which had a very high frequency of T[C > T]A (41%) and attributed to activity of the AID/APOBEC family^62^. In fact, EGFR-E762K mutation has been observed in other cancer types associated with signature 2^63^. The remaining of the top-10 mutations were associated to either C > T transitions (1 mutation in LUAD and 2 mutations in LSCC) or other nucleotide mutations (3 mutations in LSCC and 1 mutation in LUAD).

Next, EGFR mutations frequently observed in LUAD and LSCC patients were further analyzed. The T790M mutation, known to confer resistance to first-line targeted therapies in LUAD and LSCC, was ranked in positions 49^th^ and 50^th^ with a predicted likelihood of 0.015 and 0.011 in LUAD and LSCC, respectively. T790M is caused by a A[C > T]G nucleotide change, strongly associated with signature 1, which in turn correlate with age of diagnosis^40^. G719S is another EGFR mutation frequently observed in LUAD and LSCC patients. This mutation, ranked 79^th^ in LUAD with a predicted likelihood of 0.010 and 118^th^ with 0.007 likelihood in LSCC, is the result of a G[G > A]G nucleotide mutation, which has the highest probabilities in signatures 1, 6 and 16 (although the latest is not associated to LUAD). Therefore, we hypothesize that the emergence of this mutation can be associated to ageing (signature 1) and defective DNA mismatch repair (signature 6). Lack of association with signature 4 suggests that it is not particularly linked to tobacco smoking. Another interesting mutation is the recurrently reported R776H mutation, which activates EGFR in the absence of the activating EGF ligand R776H^64^,^65^. This mutation was ranked 64^th^ and 65^th^, with a predicted likelihood of 0.012 and 0.010 in LUAD and LSCC, respectively. R776H is caused by a C[G > A]C nucleotide mutation, strongly associated with signature 11. However, since this signature is not present in LUAD nor LSCC, the predicted probability value is the result of the sum of mild probabilities of C[G > A]C in signatures 1, 2, 4 and 5. Consequently, this mutation is not particularly associated with any specific mechanism of mutation. Other clinically reported mutations such as G719A or G857V appeared beyond the top 100 mutations and were not particularly associated with any signature significantly contributing to either LUAD or LSCC.

### Prediction of likely resistant EGFR mutations in gefitinib binding-site

We applied the aa-RFC to predict the resistance score of the amino acid mutations for the binding of gefitinib (Fig. 3C). There was not observed correlation between the two predicted scores (Pearson correlation coefficient = −0.05). The red area gathered a total amount of 39 *likely-and-resistant* mutations (*i.e.,* mutations that are very likely to arise and predicted to confer resistance). Examples of these mutations included M793L, G719S, H835Y, G796V, D855N, G796V or C775Y, among others. This representation allowed for the identification of those mutations with high likelihood and high resistance potential. The analysis the number of mutations and mean normalized resistance score (<NRS>) values associated to each phenotypic class revealed similar predictive trends than the observed in the original training set. A total amount of 171 mutations (46%) were predicted to belong to the RES class (<NRS> 0.52 ± 0.13). The SRES class was the second in number of predicted mutations. It had 124 mutations (35%) with an <NRS> of 0.57 ± 0.13. The ISEN class had 72 instances (19%), with an <NRS> score of 0.28 ± 0.09. None of the mutations were predicted to belong to the NEU class.

### Mapping of likelihood and resistance impact into the 3D structure of EGFR

Mapping of the amino acid accumulated resistance score and the resistance impact into the 3D structure of the EGFR kinase domain revealed the structural localization of the major players in gefitinib resistance (Fig. 3D). Residues with warmer colours represented amino acids whose mutation is more prone to decrease the gefitinib binding affinity (*i.e.,* higher resistance score), while the thickness of the ribbons represented the accumulated likelihood of that particular amino acid. The D855, localized in the DFG motif, was the amino acid with highest accumulated resistance score. More specifically, the D855A mutation was ranked as the top gefitinib-resistant mutation (1.0 NRS). D855 has been previously reported to play a major role in gefitinib binding^66^, and consequently, its mutation will likely decrease binding affinity to gefitinib. Interestingly, another D855 mutant (D855N) was ranked also within the *likely-and-resistant* mutations in LUAD (Fig. 3C). Other gefitinib-binding key residues such as L792 or M793 (both in the hinge region), were also among the top predicted mutations conferring resistance (*e.g.,* M793, which has an important main chain hydrogen bond to gefitinib) and its mutation can lead to a significant drop in gefitinib binding affinity^67^. Some M793 mutants were also included in the LUAD *likely-and-resistant* group, such as the cases of M793L or M793I (Fig. 3C). The L792P mutation in turn, will introduce the proline side chain into the hinge region of binding site. The distinctive cyclic structure of proline alongside its exceptional conformational rigidity can cause a steric clash between the proline side chain and gefitinib, with consequences for its binding. G719, localized in the phosphate-binding loop (P-loop), had several mutations among the top predicted mutants (G719V, 0.86 NRS SRES class; G719S, 0.83 NRS RES class) as well as mutations with lower predicted resistance potential (G719R 0.68 NRS, G719C 0.65, G719D 0.64 and G719A 0.63 all of them RES class) (Fig. 3C, inner panel). Specifically, to the G719S mutation, it has been previously shown that EGFR-G719S mutant, in fact, increases gefitinib binding affinity^68^. Therefore, it appears that the classification of the G719S as RES class corresponds to a false positive prediction. The factors leading to this miss-prediction could include a wrong structural modeling of the mutation, which may be unable to completely capture the important rearrangement of the P-loop, and the fact that experimentally measured cases of glycine mutations are enriched in loss of affinity (in our training set: 3 ISEN, 2 NEU, 9 SRES and 18 RES). Other mutants such as G719A/C/D/R have been also associated to increased sensitivity to TKis^69^, although results are contradictory and further confirmation is needed^70^. No G719V data associated response to gefitinib treatment was found in the literature. T790M was predicted to increase the binding affinity of gefitinib (0.35 NRS, ISEN class). This prediction contradicts initial studies suggesting that the methionine substitution in T790M led to a bulkier side chain compared to threonine and, subsequently, a greater steric hindrance to gefitinib and erlotinib binding. However, our result agrees with the mechanism of resistance proposed by Yun *et al*.^71^. They speculated that T790M causes an increment of both ATP and gefitinib binding affinity. Interestingly, the increment in affinity is not uniform for both ATP and gefitinib, which is ultimately reflected in a lower Kd/Km_[ATP]_ ratio, an estimator of inhibitory potency^71^. Similarly, the R776H mutation was also predicted to belong to the ISEN class (0.24 NRS). Experimental evidence found in the literature suggests that this mutation increases the sensitivity for TKis EGFR inhibitors^72^,^73^. A summary of the predictions and the experimental data associated with each mutation can be found in Table 1. Altogether, these results show that the aa-RFC can predict the mutation-induced phenotype, although individual interpretation of each case is required to further validate the predictions.

### EGFR-binders insensitive to the resistance-like mutations

To test whether our approach is able to systematically predict insensitive compounds to the EGFR’s *likely-and-resistant* mutations, we ran the lig-RFC predictor against all known EGFR reversible inhibitors with experimentally reported 3D structure (Fig. 3E). The gefitinib lig-RFC predictions were consistent with the predictions from the aa-RFC. The only exception found was the gefitinib-M793L, which had considerably lower value than for the aa-RFC (aa-RFC NRS 0.85, lig-RFC NRS 0.41), yet being labelled as SRES. The NRS decrease can be explained by the fact that the lig-RFC weighted more the conservation of the hydrogen bonding by the mutant leucine. Erlotinib, another FDA approved EGFR TKi used in the treatment of NSCLC malignancies, resulted in a very similar mutational profile compared to gefitinib, which agrees with pervious published data^74^.

T790M, M793L and R776H resulted in a low predicted resistant profile indicating that those mutations would confer increased sensitivity to many of the tested compounds. Conversely, other mutations, such as C775Y, resulted in a mixed profile conferring resistance to several of the tested compounds (e.g. CHEMBL2347963 or its structural analogue CHEMBL2347965) and increased sensitivity to others (e.g. CHEMBL2322330 and CHEMBL1229592). Finally, there were a total of six mutations with a highly drug-resistant profile (G796V, L792P, G719C/V, H835Y and D855A). These mutations were generally predicted as non-targetable, although a few exceptions were found. For instance, the CHEMBL1090356 compound had a NRS of 0.12, 0.20 and 0.14 for G796V, G719C/V mutations, respectively. In fact, this compound had the lowest resistance profile among all the screened set. Structural details revealed that CHEMBL1090356 has an imidazothiazole scaffold, with an amide group that lays deeply in the hydrophobic pocket and a morpholine tail that extends to a solved exposed region of the pocket^75^ (Fig. 3F). This mode of binding is significantly different to other reversible ATP-competitive inhibitors of EGFR and explains its predicted distinctive profile. We propose that this compound might be an alternative EGFR inhibitor to patients resistant to gefitinib therapy.

### ERK2 predicted mutational landscape in melanoma and colorectal cancer

**Figure 4:**
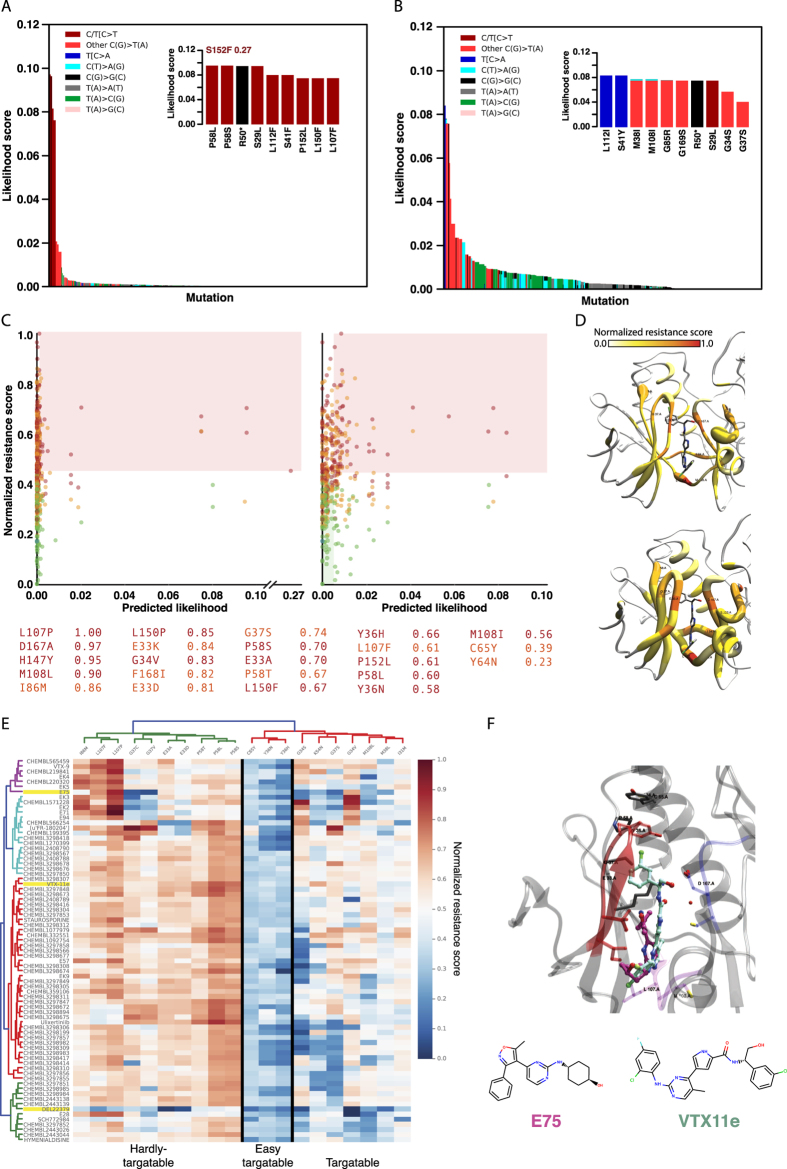
(**A**) Predicted cancer associated-likelihood of mutations in the ERK2 binding site for VTX11e for melanoma. Represented as in Fig. 3A. (**B**) Predicted cancer associated-likelihood of mutations in the ERK2 binding site for VTX11e for colorectal cancer. Representation as in panel A. (**C**) Predicted likelihood and normalized resistance score (NRS) for ERK2 mutations in the binding site of VTX11e for the melanoma (left) and colorectal (right) cancer types. Represented as in Fig. 3C. (**D**) Melanoma (top) and colorectal (bottom) mutation likelihoods and normalized resistance scores (NRS) in the 3D structure of ERK2-VTX11e complex (PDB: 4QTE). Represented as in Fig. 3D. (**E**) Predicted sensitivity map for ERK2mutations in the binding site of VTX11e. Represented as in Fig. 3E. (**F**) Structural mapping of the predicted resistance mutations in the wild type ERK2interaction with VTX11e (cyan) and E75 (magenta). PDB entries: 4QTE and 4FUX for VTX11e and E75, respectively. Represented as in Fig. 3F.

The predicted ERK2 mutational landscape revealed significant differences across the likelihood of mutations between melanoma and colorectal cancers. Indeed, the probabilities of mutations of amino acids involved in the binding site of VTX11e^76^, a compound with anti-proliferative activity, was different in melanoma^77^,^78^ and colorectal adenocarcinoma^76^ (Fig. 4A and B). Such discrepancy was the result of completely different signatures contributing to the mutational landscape. While melanomas mutations are mainly coming from C > T transitions associated to signature 7, colorectal cancer mutations are the result of multiple mechanisms associated to signatures 1, 5, 6 and 10. Melanomas predicted likelihood fitted into in a long tailed distribution, with enrichment in C > T mutations (Fig. 4A). More specifically, there were nine possible amino acid mutations originated from C/T[C > T]N changes; and all of them were ranked within the top-10 likely mutations (S153F, P58L, P58S, S29L, L112F, S41F, P152L, L150F, L107F) (Fig. 4A. inner panel). The remaining C > T mutations were also enriched among the top-50 most likely set (C > T odd ratio = 15.4). Conversely, colorectal cancer resulted in a more heterogeneous predicted mutational landscape (Fig. 4B). The two most likely mutations (L112I and S41Y) were coming from T[C > A]T mutations associated with signature 10, which has been proposed to be caused by altered activity of the error-prone polymerase POLE^79^. Furthermore, mutations resulting from C > T transitions were also enriched among the top-50 likely mutations (C > T odds ratio = 58.4). In fact, 7 out of the top-10 most likely mutations were the result C > T transitions (M38I, M108L, G85R, G169S, S29L, G34S and G37S) (Fig. 4B, inner panel). Unlike melanoma, colorectal cancer C > T mutations were associated to multiple signatures, including signatures 1, 6 and 10.

### Prediction of likely resistant mutations in ERK2-VTX11e binding-site

The resistance impact of all ERK2 amino acid mutations in the binding site of VTX11e was calculated using the aa-RFC classifier. The predictive pattern was consistent with the predictions in the training set and the EGFR case. There were 171 (40%) mutations classified as RES (0.48 NRS ± 0.15), 159 (38%) classified as SRES (0.43 NRS ± 0.14), 93 (21.9%) as ISEN (0.25 NRS ± 0.10) and 1 (0.1%) as NEU (0.17 NRS). Consistent with the observed for EGFR, the predicted likelihood and the NRS scores did no correlate (Fig. 4C, Pearson Correlation Score of 0.03 in melanoma and 0.01 in colorectal). Variations in the mutational landscape between the two cancer types were also demonstrated in the differences in the set of top *likely-and-resistant* mutations. Melanomas resulted in 79 mutations, including P58S/L/T, L150F, L107F, P152S/L, L157F, L112P, I84N, F168Y or G37S among others, as likely for the cancer type and predicted to confer resistance to VTX11e. There were 86 mutations, including G34S, G37S, H147Y, P152S, E33K, L155P, P58L/S/T or K114R among others, as likely to appear in colorectal cancer and predicted to confer resistance to VTX11e. Only 29 of the mutations were shared between the two *likely-and-resistant* groups.

### Mapping of likelihood and resistance impact into the 3D structure of ERK2

The significant differences observed between the two cancer types were also observed in the 3D mapping of the mutations into the target structure of ERK2 (Fig. 4D). Specifically, the significantly higher median likelihood observed in colorectal cancer (11.5 fold increase, colorectal median likelihood 2.6e^−3^; melanoma median likelihood 0.2e^−3^) was represented into the 3D space as thicker ribbons along the binding site of VTX11e. Similar to the EGFR case, not a particular structural pattern was observed hosting the most likely mutations. Additionally, mapping of the amino acid accumulated resistance score into the ERK2 3D structure of the ERK2 kinase domain revealed the structural localization of those residues more prone to decrease VTX11e binding affinity (Fig. 4D). Residues in the hinge region of the ATP binding-site showed the highest resistance scores. This region hosts the M108 residue, which is equivalent to the EGFR-M793, and is the major responsible of the hydrogen bonding between ERK2 and VTX11e. Examples of likely mutations of this amino acid included M108L (0.89 NRS, SRES class) and M108I (0.55 NRS, SRES class), being the later also included in the top *likely-and-resistant* group in colorectal cancer. ERK2-L107 was also predicted as one of the major contributors to resistance. Mutations of these amino acids included the L107P (1.0 NRS, SRES class) or L107F (0.41 NRS, RES class), being the later included in the *likely-and-resistant* set in melanoma. The importance of D167, localized at the DFG motif and structurally equivalent to the EGFR-D855, explains the high resistance score of the D167A mutation (NRS 0.97, SRES class). These residues were localized in the ATP-binding site of ERK2 and their potential to confer resistance might be explained by their ATP-binding site structural similarity with EGFR.

Proline 58 mutations were also classified as highly resistance-like. More specifically, P58L/S/T (0.60 NRS, 0.70 NRS and 0.67 NRS; SRES, SRES and RES class respectively) mutations were reported within the *likely-and-resistant* group in melanoma and colorectal cancer, suggesting these mutations are critical. These predictions agreed with the evidence of ERK2-P58L/S/T mutations found in VTX11e-resistant A375 melanoma cell line^80^. A complete summary of the VTX11e-resistant mutations previously described^80^ alongside their predicted likelihood and resistance-likeliness is shown in Table2 and Fig. 4C inner set. All the experimentally found resistant mutations were predicted as either SRES or RES by our model. Moreover, 5 out of 8 (62%) of the mutations were correctly predicted to belong to melanoma *likely-and-resistant* group. Altogether, these results probed the ability of the method to detect resistance-like mutations to ERK2-VTX11e interaction. Interestingly, the 3D mapping of the mutations from ref. 80 revealed their clustering into an adjacent pocket to the ERK2 ATP binding site, which highlights the presence of ATP had an essential role in the emergence of mutations conferring resistance in ATP-competitive inhibitors. Other mutations ranked in the top 10 resistance-like mutations and not present in ref. 80 included H147Y, I86M, L150P, G34V, F168I and E33D (Fig. 4C inner set). Unfortunately, no experimental data was available at the time to confirm the resistance potential of these mutants.

### ERK2-binders insensitive to the resistance-like mutations

Next, the lig-RFC classifier was applied to existing ERK2 reversible inhibitors to identify insensitive compounds to the resistance-like mutations previously identified. In this case, the limited number of co-crystallized ERK2 inhibitors, prompted us to extend the search to any known ERK2 inhibitor (see methods *Creating the dataset of candidate molecules*). Similarly to the EGFR example, some mutations had a highly resistance-like profile with a very limited number of compounds with low predicted sensitivity (Fig. 4E). Such were the cases of L107F/P, I86M or P58S/T/L; which had few compounds with NSR below the average (0.50 NRS ± 0.16). del22379 was one of the few compounds with low predicted sensitivity to highly resistant mutations. Interestingly, this compound resulted a highly insensitive profile among the all the screened mutations. DEL223790 unique sensitivity profile is explained by its completely different mode of action: it binds the ERK2 interface preventing its dimerization^81^. Other mutations resulted in low resistance impact profile, including Y36N/H or C65Y. The results of the C65Y mutation were consistent with the predictions from the aa-RFC, which scored this mutation with a low NRS (Table 2). However, the Y36N/H predictions generally resulted in lower NRS. For instance, the control compound VTX11e, resulted in a lig-RFC NRS of 0.41 (Y36N) and 0.35 (Y36H) while the aa-RFC scored them with 0.58 and 0.66. Despite of the decrease in the NRS, the predicted class was maintained in both classifiers as SRES. The differences between the two classifiers might be caused by the fact that the lig-RFC does not contain all the amino acid based features used in the aa-RFC. Finally, the G37S mutation, which had previously been identified as resistant^80^, was predicted to be in the *likely-and-resistant* group in both melanoma and colorectal cancer. G37 is localized in the ERK2 P-loop, and we hypothesize it may play an important role in the orientation of Y36 towards to the chlorobenzene group of VTX11e, which ultimately leads to the π-stacking interaction^82^. Remarkably, the lig-RFC provided several compounds with low resistance impact to G37S/V/C mutations. The compound with the lowest resistance profile for these mutations was E75 (Fig. 4F, named as E75 due to their PDB accession code). The mutational profile of E75 had a NRS of 0.11, 0.08 and 0.06 for G37S/V/C, respectively. Unlike VTX11e, E75 is located distantly to the G37 residue, not interacting with the Y36 and mostly occupying the ERK2 hinge region (Fig. 4F). Hence, the E75 binding mode might be compatible with G37 mutations, proposing an interesting candidate for overcoming resistance in tumors harboring ERK2-G37S/V/C mutations.

## Discussion

We have a novel computational framework that predicts the cancer-associated likelihood and the resistance impact of mutations in targets of small molecule targeted cancer therapies, applicable to cases where a model of the binding of the drug to the target protein is known. Our approach first defines the mutational likelihood of amino acids involved in the binding of a small molecule drug using a large set of empirically observed mutations. Our estimations rely on the tri-nucleotide mutational probabilities observed in the cancer-associated signatures previously described^36^,^40^. We have demonstrated the power of this framework to predict previously clinically described drug resistance mutants and identified novel potential mutants that can potentially infer drug resistance. We have shown that the EGFR mutational profile was not significantly different between LUAD and LSCC cancer types. Conversely, the ERK2 analysis revealed major differences between melanoma and colorectal mutational landscape. Melanoma mutations are mainly originated from C > T transitions associated to ultraviolet light exposure. However, colorectal associated mutations are the result of more complex and heterogeneous processes. Interestingly, the discrepancies are also reflected in the global distribution of the probabilities. While melanoma seems to prioritize fewer ERK2-mutations with a very high likelihood, colorectal tumors, presents a larger number of lower likelihood mutations. The differences between the colorectal cancer and melanoma mutations are also reflected in the low overlapping between the likely*-and-resistant* groups of mutations. This result suggests that clinicians treating these two cancer types should adopt different pharmacological approaches to overcome resistance due to the emerging cancer-associated mutations in the drug targets.

The nature of our approach enables the tracking of the association between each mutation and their underlying signatures, which ultimately can be translated into an individual mutation-mechanisms association. That is the case of the EGFR-T790M mutation, which we proposed to be mainly associated with ageing and not particularly linked to tobacco smoking. It is important to mention that our model only considers the probability of emergence of mutations in a cancer genomic context. Nevertheless, a significant number of mutations in a cancer cell can be also the result of germ-line variations or pre-malignant somatic mutations. For instance, it has been shown that the EGFR-T790M mutation can have both somatic and germ-line origin^83^,^84^,^85^. Another limitation of the likelihood model is that its predictions are based on the average probabilities from hundreds of samples for each cancer type. Therefore, the predicted likelihood shows global cancer trends but it is currently unable to capture specific trends in each individual cancer case. Future work might thus focus on finding the mechanisms underlying each individual cancer case, which eventually would translate into the personalization of the likelihood predictions. Of course, this method only addresses site-specific coding differences, and not many other mechanisms that give rise to cancer drug resistance, however the observation of many such site specific mutations in clinical samples highlight the importance of this mechanism.

The structural mapping of the predicted likelihood did not reveal significant association between the likelihood of an amino acid mutation occurring and its structural localization. Perhaps, constraining the mutational likelihood with evolutionary restrains would lead towards an increase in less evolutionary conserved regions of the structure. Hence, the unfavorable phenotype linked to evolutionary restraints can partially explain the fact that some of the predicted mutations have not been observed in the clinic. This problem is chiefly evident in cancer, where tumor cell population has a fitness advantage over the healthy tissue. Another explanation is linked to the technical limitations of standard NGS sequencing of solid biopsies, which only allows for the detection of mutations present >5% of tumor cells^7^. In fact, despite of tumors can harbour millions of mutations^23^, only a small percentage of them are systematically reported. These low-frequency mutations may not have a critical effect during tumor progression, but the evolutionary pressure induced by a drug treatment regimen can transform them into drug resistance drivers. Thus, it is essential to detect not only the frequent cancer drivers but also the low-frequent mutations that can lead towards drug resistance. Recent studies using circulating tumor DNA (ctDNA) have shown very promising results for this purpose^86^,^87^. However, there are many technological challenges to address prior to broader application of this technology. In the meantime, *in-silico* models can play a major role to comprehensively characterize the mutational burden of cancer samples.

We connected the mutational landscape of tumors with the drug-resistance phenotype due to spontaneously generated mutations in drug targets. To do so, the aa-RFC classifier predicts the effect of a single mutation to the drug binding affinity in a particular cancer target. The classifier was trained with the Platinum database^46^, whose instances were split into four phenotypic classes depending of their drug binding affinity fold change. In our opinion, reducing the number of possible classes from four to two (*e.g.* into *loss-of-affinity* and *gain-of-affinity)* would increase the classifier performance, but it would also over simplify the spectrum of possible phenotypes. Evaluation of the performance of the aa-RFC showed that classes representing severe changes (*i.e.,* ISEN and SRES classes) outperformed those representing mild changes (*i.e.,* RES and NEU classes). More specifically, the lower performance of these classes is the result of over prediction towards the RES class as well as under prediction of the NEU class. This limitation may be explained by the fact that many RES cases are very close to the NEU frontier (*i.e.,* cases with very small drop in affinity) and vice versa. In such cases, the classifier assigns the instances to the most populated class (*i.e.,* the RES class) since that is the one with higher probability. To address this limitation, we calculated the NRS, which provides a smoother way to assess the resistance impact by combining the confidence score of the four classes and correcting for the over-assignment of the most populated classes. To our knowledge this is the first method specifically developed to classify changes in drug binding affinity upon mutation. Comparison with gold-standard methods for measuring drug-binding affinity revealed the difficulties of such methods in detecting large changes in affinity upon mutation. Rather, they are oriented to quantitatively estimate the drug binding affinity when the binding is known to occur.

Application of the aa-RFC to the EGFR and ERK2 cases showed its ability to identify the phenotype of previously reported mutations. Remarkably, the method correctly predicted the class of EGFR-T790M, conferring resistance by decreasing the Kd/Km_[ATP]_ ratio; EGFR-R776H, ERK2-P58L/S/T or ERK2-G37S among others. However, it failed predicting the EGFR-G719S phenotype, which featured the problem that glycine mutations increasing the sensitivity of the drug are likely to be miss-classified. Additionally, our model proposed, in both cases, multiple new unseen mutations as candidates for conferring resistance to the studied treatments. Nevertheless, mutations negatively interfering with ATP might be non-functional. Mutations disrupting the ATP binding would lead to a non-functional protein kinase (*i.e.,* loss of function mutations), which is usually incompatible with their role in cancer progression. This hypothesis is also supported by previous findings indicating a cluster of ERK resistant mutations in an allosteric region next to the ATP binding site^80^. Moreover, our findings might explain why mutants of amino acids with an essential role in ATP binding, such as EGFR-L792 (ERK2-L107) or EGFR-M793 (ERK2-M108), have not yet been reported in the clinics. Examination of public large-scale cancer genomic data does not reveal many of these mutations (Tables 1 and 2 and supplementary information). This is primarily because these data focus on primary untreated tumors, and resistance mutants are likely to be of extremely low tumor frequency in these datasets.

Importantly, this model only focuses on drug resistance arising from single point mutations affecting the binding of small molecule targeted cancer therapies. However, as mentioned in the introduction, there are numerous alternative mechanisms responsible of drug resistance. For instance, there is an emerging evidence of kinase mutations not directly interfering with the drug and yet having an impact in the drug response. These mutations may drive resistance by enhancing other non-enzymatic kinase functions that may be equally important for tumor progression. For instance, in melanoma and colorectal cancer, tumors harbouring K-RAS, H-RAS, N-RAS or B-RAF constitutively active mutations may be insensitive to ERK1/2 inhibitors. In such cases, combinatorial regimes (e.g. B-RAF and ERK inhibitors^88^) might be an alternative to overcome resistance.

The last step of the model application consisted on the search for non-resistant molecules to the mutations detected by the aa-RFC. To do so, we used a lighter and more ligand centric version of the aa-RFC called lig-RFC. The performance of both classifiers is also illustrated by the consistency of the EGFR-gefitinib and ERK2-VTX11e predictions. However, small discrepancies in the NRS score were observed for the ERK2-Y36H/N and EGFR-M793L mutations. In both cases the differences respond to the fact that the lig-RFC weights more the ligand-based features (*e.g.*, hydrogen bonding conservation) that changes in the amino acid biochemical properties. An important limitation is that the model only applies to non-covalent reversible inhibitors, and so wouldn’t cover resistance to say, for example third generation irreversible EGFR inhibitors, however, in these cases simple rule-based systems will be highly predictive.

In both, ERK2 and EGFR cases, we observed three groups of mutations. The first group, named as *hardly targetable*, was composed by those mutations with very limited or no compounds with low resistance score. That is, it encompasses mutations that are generally predicted as non-targetable by our method. However, some exceptions were found within this group. Exceptions included the dimerization inhibitor del22379 predicted to be insensitive to the majority of the ERK2-mutations and CHEMBL1090356, which was predicted as insensitive to most of the non-targetable mutations of EGFR. These two cases were the result of very distinctive mode of actions, which ultimately was reflected in their resistance profile. The second group of mutations, named as *easily targetable*, was composed by those mutations predicted to increase the affinity of most of the screened compounds. Interestingly, despite of EGFR-T790M being known to confer resistance to most of EGFR reversible inhibitors, it was classified into this group of mutations. This is because this mutation confers resistance by decreasing the Kd/Km_[ATP]_ ratio. The third group of mutations, named as *targetable*, was composed by mutations predicted to have heterogeneous resistance profile across the screened compounds. This group is probably the most interesting from a resistance perspective, since they allowed the study of the structural differences that might be driving the emergence of resistance. For instance, in the ERK2-G37S example, we observed how the low occupancy of the allosteric pocket posited E75 as an interesting candidate to overcome resistance due to mutations occurring in this region.

Future applications of the model would benefit from the inclusion of both new candidate molecules and information about resistant mutants. Moreover, further application in other systems would ultimately lead towards a comprehensive characterization of the resistant mutational landscape of targeted cancer therapies. To achieve this goal, it is also important that the scientific community validates some of our predictions. Despite of screenings of low-frequency mutations may be not cost-effective due to the limited amount of patients benefiting from such stratification, advances in screening technologies, patient-derived tumor xenograph and computational models may help to mitigate the expenses associated to these screenings. All these advances would eventually get closer the desired goal of tailored design of non-resistant cancer therapies.

## Additional Information

**How to cite this article:** Martínez-Jiménez, F. *et al*. Rational design of non-resistant targeted cancer therapies. *Sci. Rep.* **7**, 46632; doi: 10.1038/srep46632 (2017).

**Publisher's note:** Springer Nature remains neutral with regard to jurisdictional claims in published maps and institutional affiliations.

## Supplementary Material

Supplementary Information

Supplementary Dataset 1

Supplementary Dataset 2

Supplementary Dataset 3

Supplementary Dataset 4

Supplementary Dataset 5

Supplementary Dataset 6

Supplementary Dataset 7

Supplementary Dataset 8

Supplementary Dataset 9

Supplementary Dataset 10

Supplementary Dataset 11

Supplementary Dataset 12

Supplementary Dataset 13

## Figures and Tables

**Table 1:** Summary of the EGFR mutations discussed in the manuscript alongside their aa-RFC predicted phenotype and, when available, the experimentally reported effect to gefitinib treatment found in literature.

Mutation	NMR	Predicted class	Gefitinib phenotype	Reported in TCGA
L792P	0.88	SRES	Proposed resistant (Unconfirmed)	No
M793L	0.85	SRES		No
D855N	1.0	SRES		No
G719S	0.83	RES	Increase Sensitivity to gefitinib and erlotinib	LUAD
G719A	0.63	RES	Contradictory	LUAD
G719R	0.68	RES		No
G719C	0.65	RES		No
G719D	0.64	RES		No
G719V	0.86	SRES		No
T790M	0.34	ISEN	Increase Sensitivity gefitinib and erlotinib	LUAD
R776H	0.24	ISEN	Increase Sensitivity gefitinib and erlotinib	LUAD
C775Y	0.66	SRES	Unknown	No
H835Y	0.84	SRES		No
G796V	0.85	SRES		No

**Table 2:** Predicted aa-RFC phenotype of the ERK2-VTX11e resistant mutants reported in ref. 80.

Mutation	NMR	Predicted class	Top Likely-and-resistant Melanoma?	Top likely-and-resistant Colorectal?	Reported in TCGA
P58L	0.60	SRES	YES	YES	No
P58S	0.70	SRES	YES	YES	No
P58T	0.67	RES	YES	YES	No
G37S	0.74	RES	YES	YES	No
Y64N	0.23	RES	NO	NO	No
Y36H	0.66	SRES	YES	NO	No
Y36N	0.58	SRES	NO	NO	No
C65Y	0.38	RES	NO	NO	No

The top *likely-and-resistant* columns indicate their presence among the mutations included in the red area from Fig. 4B and C.
