# Supplementary material for: Rational design of non-resistant targeted cancer therapies
Source: Sci Rep. 2017 Apr 24;7:46632. doi: 10.1038/srep46632 (PMC5402386; doi:10.1038/srep46632)
Supplement: Supplementary Information [file srep46632-s1.pdf]

Rational design of non-resistant targeted cancer therapies.

Francisco Martínez-Jiménez[1,2,3,], John P. Overington[4], Bissan Al-Lazikani[5] and Marc A. Marti-Renom[1,2,3,6,*]

1. CNAG-CRG, Centre for Genomic Regulation (CRG), Barcelona Institute of Science and Technology (BIST), Baldiri i Reixac 4, 08028 Barcelona, Spain.
2. Gene Regulation, Stem Cells and Cancer Program, Centre for Genomic Regulation (CRG), Dr. Aiguader 88, 08003 Barcelona, Spain.
3. Universitat Pompeu Fabra (UPF), Barcelona, Spain.
4. Benevolent.ai, 40 Churchway, London NW1 1LW, UK.
5. The Institute of Cancer Research, London, UK.
6. ICREA, Pg. Lluís Companys 23, 08010 Barcelona, Spain.

SUPPLEMENTARY INFORMATION

contribution_cancer_signatures.txt
File with the calculated contributions of each signature for each cancer class. Data calculate from
matrix_EGFR.csv
Raw data from figure's 3 matrix.
matrix_ERK2.csv
Raw data from figure's 3 matrix.
original_single_dataset.csv
Original platinum dataset after removing instances including more than 1 mutation.
original_single_dataset_features_aarfc.csv
aa-RFC features calculated for the original platinum dataset.
original_single_dataset_features_ligrfc.csv
lig-RFC features calculated for the original platinum dataset.
prediction_EGFR_LSCC.tsv
Complete list of aa-RFC predictions for al EGFR-gefitinib binding site aa mutations in LUAD.
prediction_EGFR_LUAD.tsv
Complete list of aa-RFC predictions for al EGFR-gefitinib binding site aa mutations in LSCC.
prediction_ERK2_colorectal.tsv
Complete list of aa-RFC predictions for al ERK2-vtx11e binding site aa mutations in colorectal adenocarcinoma.
prediction_ERK2_melanoma.tsv
Complete list of aa-RFC predictions for al ERK2-vtx11e binding site aa mutations in melanoma.
training_aarfc_balanced.csv
Final aa-RFC training set after balancing the original platinum dataset.
training_ligrfc_balanced.csv
Final lig-RFC training set after balancing the original platinum dataset.
signatures.txt
192x30 matrix representing the trinucleotide mutational probabilities associated to each of the 30 signatures.
